# Evolutionary Systems Biology of Amino Acid Biosynthetic Cost in Yeast

**DOI:** 10.1371/journal.pone.0011935

**Published:** 2010-08-17

**Authors:** Michael D. Barton, Daniela Delneri, Stephen G. Oliver, Magnus Rattray, Casey M. Bergman

**Affiliations:** 1 Faculty of Life Sciences, University of Manchester, Manchester, United Kingdom; 2 Department of Biological Sciences, Northern Kentucky University, Highland Heights, Kentucky, United States of America; 3 Department of Biochemistry, University of Cambridge, Cambridge, United Kingdom; 4 School of Computer Science, University of Manchester, Manchester, United Kingdom; University College London, United Kingdom

## Abstract

Every protein has a biosynthetic cost to the cell based on the synthesis of its constituent amino acids. In order to optimise growth and reproduction, natural selection is expected, where possible, to favour the use of proteins whose constituents are cheaper to produce, as reduced biosynthetic cost may confer a fitness advantage to the organism. Quantifying the cost of amino acid biosynthesis presents challenges, since energetic requirements may change across different cellular and environmental conditions. We developed a systems biology approach to estimate the cost of amino acid synthesis based on genome-scale metabolic models and investigated the effects of the cost of amino acid synthesis on *Saccharomyces cerevisiae* gene expression and protein evolution. First, we used our two new and six previously reported measures of amino acid cost in conjunction with codon usage bias, tRNA gene number and atomic composition to identify which of these factors best predict transcript and protein levels. Second, we compared amino acid cost with rates of amino acid substitution across four species in the genus *Saccharomyces*. Regardless of which cost measure is used, amino acid biosynthetic cost is weakly associated with transcript and protein levels. In contrast, we find that biosynthetic cost and amino acid substitution rates show a negative correlation, but for only a subset of cost measures. In the economy of the yeast cell, we find that the cost of amino acid synthesis plays a limited role in shaping transcript and protein expression levels compared to that of translational optimisation. Biosynthetic cost does, however, appear to affect rates of amino acid evolution in *Saccharomyces*, suggesting that expensive amino acids may only be used when they have specific structural or functional roles in protein sequences. However, as there appears to be no single currency to compute the cost of amino acid synthesis across all cellular and environmental conditions, we conclude that a systems approach is necessary to unravel the full effects of amino acid biosynthetic cost in complex biological systems.

## Introduction

Everything in a living cell has a cost: from the energy needed to transform molecules against thermodynamic equilibria, to the raw materials needed to produce the constituents of a new cell. Natural selection may be expected to minimise such cellular costs, and evidence for adaptation to require less energy or matter may exist at the molecular or cellular level. This theory is described as the cost minimisation hypothesis. Testing this hypothesis requires answering several questions about what is the meaning of cost in the cell, and how best to measure it. For example, how does one assign a biochemical price to a molecule whose state is dependent on changing environmental and cellular conditions? Similarly, is it possible to understand the energetic costs required for metabolite synthesis, independent of other cellular functions? Knowing the answers to these questions is central to a systematic understanding of the chemical forces that shape the composition of biomolecules, and how biomolecular composition relates to protein expression and evolution.

Craig and Weber [1] pioneered the quantitative analysis of cost at the cellular level to investigate the effects on the synthesis and evolution of a small number of *Escherichia coli* proteins. These authors estimated the cost of a protein as the per-residue average of how many units of high energy phosphate bonds (e.g. ATP) and reducing hydrogen atoms (e.g. NADPH) are diverted from the available energy pool to produce each of the constituent amino acids from glucose. Akashi and Gojobori [2] used a modified version of this approach to show in *E. coli* and *Bacillus subtilis* that predicted gene expression levels based on codon usage bias show a negative correlation with average protein cost. This work provided the first genome-wide evidence that evolution has optimised prokaryotic cells to use less expensive amino acids in highly expressed proteins and established an important link between the metabolism of a cell and the evolution of its genome sequence.

Heizer *et al.* [3] extended the findings of Akashi and Gojobori [2] to four additional prokaryotic species and demonstrated that metabolic cost optimisation occurs whether the source of energy is organic or inorganic. Swire [4] used Craig and Weber's [1] cost values to generate a new cost measure for an amino acid based on its usage in proteins as a function of overall protein cost computed from all other amino acids, and showed that cost selection affects multiple prokaryotic, archaeal and eukaryotic genomes. Wagner [5] developed a method similar to Craig and Weber [1] that includes the energetic costs of synthesising both mRNA and protein for *Saccharomyces cerevisiae*, and showed that the cost of doubling gene expression after a gene duplication is likely to be significant enough to be detected by natural selection. More recently, Raiford *et al.* [6] compared Wagner's [5] amino acid biosynthetic costs with codon bias, transcript levels and protein levels and observed that cost minimisation is observed across different functional categories of genes, but that its effects are restricted to certain amino acids.

Seligmann [7] argued that, while the number of high energy molecules is an important part of the energetic investment of synthesising an amino acid, this approach is unlikely to explain the entire investment made by a cell. Instead, Seligmann [7] used the molecular weight as a proxy for biosynthetic cost, reasoning that this may take into account all the manifold effects of producing larger, more complex amino acids. Molecular weight also has the advantage of being constant across species, and therefore can be used to test the cost selection hypothesis where the genome sequence is available but the pathways for amino acid synthesis are unknown. Seligmann [7] used this to prove, on an individual protein basis, that molecular weight is minimised across a range of bacterial and eukaryotic genomes.

Despite the widespread conservation of amino acid biosynthetic pathways, using a fixed set of energetic requirements may not represent the true cost of amino acid synthesis in different cellular and environmental conditions. For example, Wagner [5] has shown that the estimated cost of amino acid biosynthesis varies under different modes of growth (fermentative vs. respiratory). The cost of amino acid synthesis may also vary as a function of limiting nutrients in the environment. Thus, developing methods to investigate the cost of amino acid synthesis under varying environmental conditions is essential to understanding the impact of biosynthetic cost on cellular systems. Just as in supply and demand economics, when a chemical resource is scarce in the cell or environment, synthesis of biomolecules that require this resource will be more expensive in comparison to molecules where that resource is utilised less [8]. As an example of the effect of supply and demand in cellular economics, Varma *et al.* [9] showed in *E. coli* that the “shadow price” of using molecules involved in energy production changes according to the availability of oxygen. As the availability of oxygen decreases, the cost of its use rises, while the cost of ethanol use decreases as the energy to reduction-oxidation ratio becomes less efficient. Likewise, Carlson [8] demonstrated a similar principle of supply and demand by showing *in silico* that *E. coli* will likely favour the use of cheaper, but less efficient, pathways in stress inducing environments.

In this report, we use a systems biology approach based on flux balance analysis (FBA), similar to methods of Varma *et al.* [9], to estimate the cost of synthesising amino acids under differing nutrient availabilities in the environment. We first estimate the “absolute” cost of amino acid synthesis by examining the sensitivity of nutrient uptake in the cell to small absolute changes in the stoichiometric requirement of each amino acid for growth in the FBA model. We then estimate a second “relative” cost by examining the sensitivity of nutrient uptake to small percentage changes in the amino acid stoichiometric requirement. We calculate each of these amino acid cost types for three nutrient limiting conditions (glucose, ammonia and sulphate) to investigate how cost varies from environment to environment. We focus our analysis on protein coding genes, because as in previous studies [1], [3]–[5], [7] this allows us to analyse the effects of biosynthetic cost at the transcript and protein levels, which we extend here to the analysis of rates of amino acid substitution across species. The results in this work show that biosynthetic cost has a small but measurable relationship with transcript and protein levels, independent of codon usage bias, per codon tRNA gene number or protein atomic content. Furthermore, we show that biosynthetic cost is negatively correlated with rates of amino acid evolution and conclude that selection for cost minimisation does indeed play a role in *Saccharomyces* protein expression and evolution.

## Results

### A systems biology approach to estimating the cost of amino acid synthesis

Our analysis uses systems biology to determine the cost of an amino acid using a model of metabolism that takes into account many of the requirements for *S. cerevisiae* cellular growth. This analysis is based on *in silico* reconstructions of the reaction networks comprising cellular metabolism in organisms such as *E. coli* and *S. cerevisiae*. These ‘genome scale models’ are formulated as a matrix 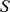 that describes the connectivity between the hundreds of reactions in metabolism. Organism metabolic phenotypes can then be simulated using these models combined with a technique called flux balance analysis (FBA) reviewed in [10]. FBA uses the matrix of reactions in a genome scale model to find the optimal combination of reactions that consume available nutrients (e.g. glucose and ammonium) to produce the metabolites (e.g. sugars, fats, high energy molecules) required for new cellular growth (also described as biomass production).

Our approach to estimating biosynthetic cost simulates small changes in the demand for an amino acid in cell growth (as part of the biomass producing reaction) then analyses the corresponding response in the supply of three nutrients: glucose, ammonium and sulphate. To simulate a change in amino acid demand, the *in silico* requirement for each amino acid was changed by a range of small values around the original requirement defined in the model. Each change added a slight increase or decrease in the requirement of the amino acid for biomass production. Amino acid requirements were changed at the 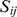 position in the model stoichiometric matrix, with position 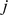 corresponding to the reaction producing new biomass components for growth and 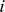 corresponding to the amino acid being examined. For each change in amino acid requirement, FBA was used to simulate the smallest possible uptake flux of either glucose, ammonium, or sulphate entering the cell whilst still maintaining the fixed growth rate. The vector of changes in amino acid requirement produced a corresponding vector for the effect each change had on nutrient entry into the cell. The minimisation of each nutrient entry into the cell aimed to simulate a limiting environment for that particular nutrient (e.g. glucose, where all other nutrients including ammonium and sulphate are in abundance). In our approach, the biomass reaction is fixed to a constant growth rate and then a specific uptake flux is minimised. It is of course possible to do the reverse, i.e. fix the uptake of a specific nutrient and then optimise the biomass flux. The reason we adopted the fixed growth rate approach is because this simulation framework mimics the continuous culture of a chemostat, which is how experimental data on gene expression are often produced. A useful side effect though is that all simulations are performed on the same growth rate, which makes them more easily comparable because they are on the same scale (see below).

Using this approach, two types of amino acid cost were estimated. The first type was derived from changing each amino acid requirement by an absolute amount around the original value, which we refer to as “absolute” amino acid cost. A second cost was calculated by changing amino acid requirement by a relative percentage around the original value, which we refer to as “relative” amino acid cost. The relationship between absolute and relative costs is formally shown in the Methods. In both cases, the cost of the amino acid was then estimated from the slope of the simulated effect on nutrient uptake as a function of changing the amino acid requirement. The greater the change in nutrient supply with change in amino acid requirement, the steeper the slope, and therefore the more “expensive” the amino acid. Absolute and relative costs were computed in three simulated nutrient limitations, providing us with six estimates of amino acid cost. To assist in the discussion of these costs we adopted the convention of 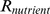 and 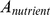 for either relative or absolute costs in each simulated nutrient limitation.

One important consideration for our approach is the model growth rate at which each cost type was estimated. A common objective when performing FBA is to determine the maximum possible growth rate for a genome scale model. Here we instead aimed to simulate nutrient limitation rather than maximum growth rate. Therefore, we fixed growth rate to a constant value and found the FBA solution for the minimum nutrient entry in the cell. To address the potential dependence of cost on the growth rate constant, we estimated each amino acid cost at a range of feasible yeast growth rates (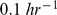, 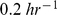 and 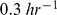) [11]. We indeed found that each cost estimate was proportional to the growth rate constant at which it was estimated, but that this dependency could be removed by dividing the cost estimate by the growth rate at which it was estimated. Using this rescaling, the variation in cost estimates observed for each cost type across growth rates was low, with the largest difference in rescaled costs over the three model growth rates being 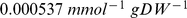 for the phenylalanine 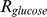 cost, and 0.0145 for the tryptophan 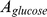 cost. These results indicate that our rescaled cost estimates are largely unaffected by model growth rate. For the purpose of this study, we used the amino acid costs estimated at a growth rate of 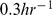.

### Comparison of systems biology derived biosynthetic costs with previous estimates

**Figure 1:**
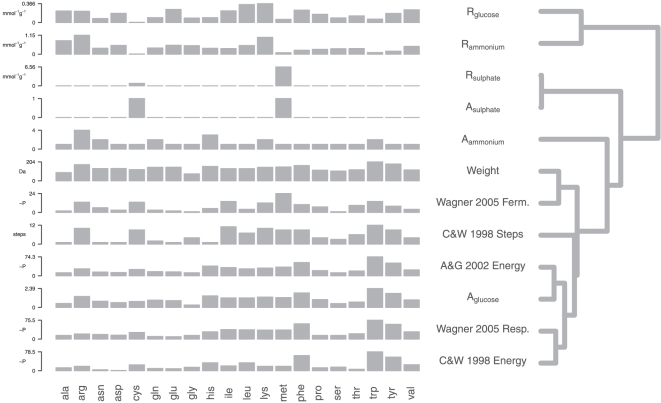
Comparison of amino acid biosynthetic cost estimates. Amino acid biosynthetic cost estimates are compared in the barcharts on the left hand side. Similarities among different cost types are visualised as a tree on the right hand side. The closer two cost types are in the tree the more similar the cost estimates. Each barchart axis shows the minimum and maximum value of each cost type, rounded to three significant figures. We note that absolute costs computed using our systems biology approach are unitless (see Materials and Methods for details). The cost comparison dendrogram was generated using complete agglomerative clustering of Spearman's Rank correlations between each cost type (see File S2).

Amino acid biosynthetic costs estimated using our systems biology method along with those reported previously in the literature are compared in Table 1 and along the left hand side of Figure 1. The right hand side of Figure 1 shows a dendrogram visualising the similarity between our estimated costs with those previously reported in the literature based on an agglomerative hierarchical clustering of pairwise Spearman's rank correlations between each amino acid cost type. All cost types used in this analysis are available in Supplementary File S1. The Spearman's *R* and *p*-values for the correlations between costs are available in Supplementary File S2.

**Table 1:** Measures of amino acid cost.

	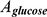	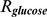	A&G Energy	C&W Energy	C&W Steps	Wager Ferm.	Wagner Resp.	Seligmann Weight
ala	0.50	0.223	11.7	12.5	1	2	14.5	89.1
arg	1.39	0.218	27.3	18.5	10	13	20.5	174.2
asn	0.79	0.078	14.7	4	1	6	18.5	132.1
asp	0.61	0.178	12.7	1	1	3	15.5	133.1
cys	0.75	0.005	24.7	24.5	9	13	26.5	121.2
gln	0.92	0.095	16.3	9.5	2	3	10.5	146.2
glu	0.86	0.254	15.3	8.5	1	2	9.5	147.1
gly	0.31	0.087	11.7	14.5	4	1	14.5	75.1
his	1.46	0.094	38.3	33	1	5	29	155.2
ile	1.21	0.226	32.3	20	11	14	38	131.2
leu	1.21	0.348	27.3	33	7	4	37	131.2
lys	1.31	0.366	30.3	18.5	10	12	36	146.2
met	1.25	0.062	34.3	18.5	9	24	36.5	149.2
phe	1.84	0.240	52.0	63	9	10	61	165.2
pro	0.99	0.159	20.3	12.5	4	7	14.5	115.1
ser	0.49	0.089	11.7	15	3	1	14.5	105.1
thr	0.69	0.128	18.7	6	6	9	21.5	119.1
trp	2.39	0.066	74.3	78.5	12	14	75.5	204.2
tyr	1.77	0.176	50.0	56.5	9	8	59	181.2
val	0.96	0.246	23.3	25	4	4	29	117.2

Rescaled 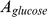 cost is unitless and 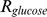 cost is in units of 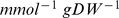. The Akashi and Gojobori [2], Craig and Weber energy [1], and the Wagner fermentative and respiratory costs [5] are based on curation of the number of high-energy molecules used during synthesis, converted to potential high energy phosphate bonds. The Craig and Weber ‘steps’ measure [1] is based on the number of biosynthetic steps between central metabolism and the resulting amino acid. Amino acid molecular weight as used by Seligmann [7] is measured in Daltons. All cost estimates are available in Supplementary File S1.

Under conditions simulating glucose limitation, our 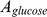 cost is clearly correlated with other previously reported measures of amino acid cost. 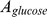 has Spearman correlation coefficients greater than 0.8 with Akashi and Gojobori's energetic cost [2], Craig and Weber's energetic cost [1], Wagner's respiratory energetic cost [5], and molecular weight [7] (Supplementary File S2). Wagner's fermentative energy cost [5] and Craig and Weber's biosynthetic complexity [1] show lower correlation coefficients of 0.522 and 0.65, respectively. These results indicate 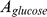 is in good agreement with estimates based on manually-curated measures previously described in the literature. In contrast, our 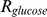 cost shows no significant correlation with any previously described cost measure (all 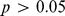). The highest Spearman coefficient between 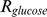 and any other literature dataset is 0.077 (*p* = 0.49, with Wagner's fermentative energetic cost [5]). This indicates that our 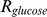 cost has little in common with previous formulations of amino acid cost.

Under conditions simulating ammonium- and sulphate-limitation, we find that the 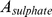 and 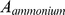 costs are directly proportional to the amount of either nitrogen or sulphur atoms in the amino acid, respectively. In contrast, the 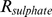 and 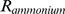 costs reflect both the composition of either sulphur or nitrogen in the amino acid and the usage of the amino acid in the reaction producing new biomass for cell growth. The contrast between the absolute and relative estimates of amino acid cost under these conditions can be illustrated by the costs for cysteine and methionine. These two amino acids both contain a single sulphur atom and therefore the 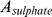 cost of each is the same: one. The 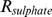 cost of methionine however is much greater than that of cysteine as the proportional use of methionine in the biomass reaction is greater. One observation of potential interest is that the 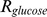 and 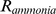 costs are correlated (Spearman 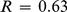, 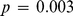). We speculate that this correlation may reflect an intrinsic adaptation in amino acid biosynthetic pathways, as any mutations to minimise biosynthetic cost under glucose limitation would also minimise the cost of amino acid synthesis in an ammonium-limited environment.

### Comparison of costs in *E. coli* and *S. cerevisiae*

Given the relative ease with which amino acid costs can be calculated using our systems biology method, we estimated 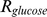 and 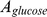 costs for *E. coli* using the iJR904 model [12] (Supplementary File S3). The aim of this was to demonstrate the generality of our approach and to explore the similarity of amino acid biosynthetic cost estimates across species and FBA models. Our analysis showed the 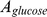 costs are highly correlated between species (Spearman 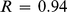, 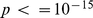), as are 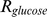 costs (Spearman 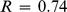, 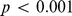). The higher correlation of 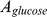 costs is expected given the conservation of core metabolic pathways across species [13], whereas the greater variation in 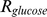 costs may arise from species specific variation in amino acid usage. Overall this demonstrates the general applicability of our method to any species with a genome-scale metabolic model.

### The cost of amino acid synthesis on the yeast transcriptome and proteome

#### Gene expression at the transcript level

If biosynthetic cost is a selective force acting on cells, we expect to observe a negative correlation between the biosynthetic cost of the encoded protein and gene expression levels [2], [6]. Therefore, we investigated the capacity of 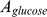 and 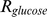 cost (as well as each previously reported cost measure) to explain gene expression at the transcript level in the *S. cerevisiae* dataset of Castrillo *et al.* [11] using multivariate regression. The expression of each transcript was modelled as a function of the mean energetic cost per residue of the protein, the codon usage bias of the coding sequence measured by the codon adaptation index (CAI), mean number of tRNAs per residue, and the mean atomic composition per residue of the protein. Since codon usage and tRNA number are known to correlate with each other [14], and codon usage is known to correlate with both gene expression [14], [15] and cost [2], their joint inclusion in our model allows us to demonstrate an independent effect of cost that controls for these factors. For this analysis, we only investigated the explanatory power of our cost measures estimated under glucose limiting conditions, as this is the environment thought to be most relevant to yeast biology [11], [16], [17]. Table 2 shows the explanatory power for the full multivariate regression for each cost type in predicting transcript levels. All regression models explain 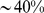 of the variation in transcript levels across genes, with the difference in variation explained by the best and worst being only 4.5%.

**Table 2:** Adjusted 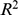 coefficients for multiple regression models.

Cost type	Transcripts Castrillo *et al*. 2007	Proteins Ghaemmaghami *et al.* 2003
*S. cerevisiae * 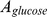	0.389	0.406
*S. cerevisiae* 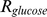	0.383	0.408
Akashi & Gojobori (2002)	0.398	0.405
Craig & Weber (1998) Energy	0.416	0.40
Craig & Weber (1998) Steps	0.375	0.404
Wagner (2005) Respiratory	0.382	0.405
Wagner (2005) Fermentative	0.377	0.406
Molecular Weight	0.422	0.405

The 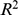 describes the fit of each regression with CAI, tRNA gene number, atomic content, and biosynthetic cost explain variation in experimental data. Each row represents the specific cost estimate used in that regression.

**Figure 2:**
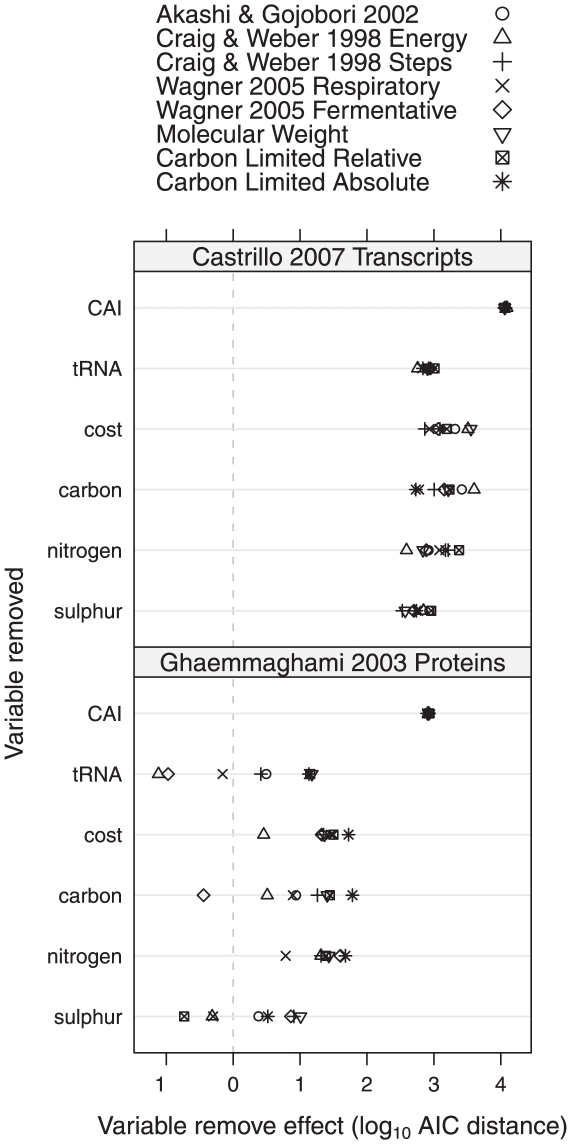
Comparison of factors explaining observed transcript and protein levels in *S. cerevisiae*. Each point is the contribution of the variable to explaining either protein or transcript levels. Points to the right have a greater contribution and vice versa for points to the left. Multiple points are shown for each variable in the figure, one for each cost type used in separate regression models fitted to explain transcript and protein levels using the following explanatory variables: average per residue protein carbon, nitrogen, sulphur content, average per residue protein biosynthetic cost, average per codon tRNA gene number and transcript CAI. The contribution of each variable to explaining transcript and protein data was then estimated by removing the variable from the regression and then estimating the size of the effect on explanatory power measured using Akaike's Information Criterion (AIC).

Using Akaike's Information Criterion (AIC) [18] the importance of the variables in the regression equation was measured by removing each in turn, then comparing the goodness-of-fit of the reduced model with the full model containing all terms. Figure 2A compares the importance of each variable in explaining transcript levels with other variables in the same regression model for each cost type. Compared to other characteristics of the encoded protein, the codon bias of the transcript is at least half an order of magnitude more important than the nearest explanatory variable, regardless of which cost type is used. This result supports the well-established fact that codon bias correlates with gene expression levels in growing yeast cells [14], [15], [19], [20]. The dominant influence of codon bias over other factors also explains why the use of different cost types in the multivariate regression model does not substantially effect the predictive power. A general trend across all the regression analyses is that the most important variable after codon bias is either biosynthetic cost, carbon content or nitrogen content. The importance of tRNA number on transcript levels appears relatively constant regardless of which cost is used. Finally sulphur content appears the least predictive measure of transcript levels.

#### Gene expression at the protein level

The importance of biosynthetic cost in explaining gene expression at the protein level was also assessed using multivariate regression followed by variable removal using the same strategy as for transcript levels above. To analyse the effect of cost on protein levels, we used data from Ghaemmaghami *et al.* [21], since protein levels from Castrillo *et al.* [11] were measured relative to a background (see Methods for details). Table 2 illustrates the explanatory power of each regression model to predict protein levels. As with the transcript data, each regression model explains 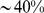 of the variation in protein levels, and the difference in variance explained between the best and worst model is very small (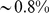), relative to the overall variance explained.

Figure 2B shows the relative importance of each factor in the multivariate regression model for protein expression levels. This analysis reveals similar trends to that for transcript levels where codon bias is the most important factor by an order of magnitude. This result is not surprising given that Ghaemmaghami *et al.* [21] previously showed a Spearman's rank correlation of 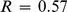 for the relationship between CAI and protein abundance. The best regression fit uses 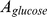, in which biosynthetic cost, carbon content and nitrogen content all have a similar importance in explaining variation in protein levels. Protein levels exhibit similar trends to transcript levels where generally (i.e. across all regression models) biosynthetic cost, carbon content and nitrogen content all play a similar importance in explaining variation in gene expression levels, and sulphur content is the least important. However the importance of tRNA number and sulphur content are more variable in explaining protein expression levels and in some instances their removal improves model parsimony, as indicated by a reduced AIC.

### Biosynthetic cost trends in protein sequence relative substitution rates

In addition to investigating the potential effects of amino acid biosynthetic cost on transcript and protein levels, we considered whether cost minimisation may also affect rates of amino acid substitution across yeast species. Specifically, we sought to test if rates of amino acid substitution at a particular site in a protein showed a negative correlation with amino acid cost, which might be expected under the cost selection hypothesis if costly amino acids used at structurally or functionally important sites are conserved in evolution.

The rate of amino acid substitution per site was estimated at each position in alignments of protein sequence from four *Saccharomyces* species for 3334 genes. Substitution rates at each site were divided by the estimated tree length across the entire gene to control for the mean substitution rate of the encoding gene. This rescaling was performed to control for factors that affect the rate of protein evolution that act at the level of the gene (e.g. expression level). The single gene alignment with an estimated tree length of 0 was removed from the analysis. This resulted in 1.66 million substitution rate estimates, one at each individual alignment column. In addition to the alignment column substitution rate, the ancestral amino acid at each site was also predicted. The mean substitution rate was calculated for each amino acid across all sites where it was inferred in the ancestral protein and then compared with the *S. cerevisiae* biosynthetic cost for that amino acid.

**Figure 3:**
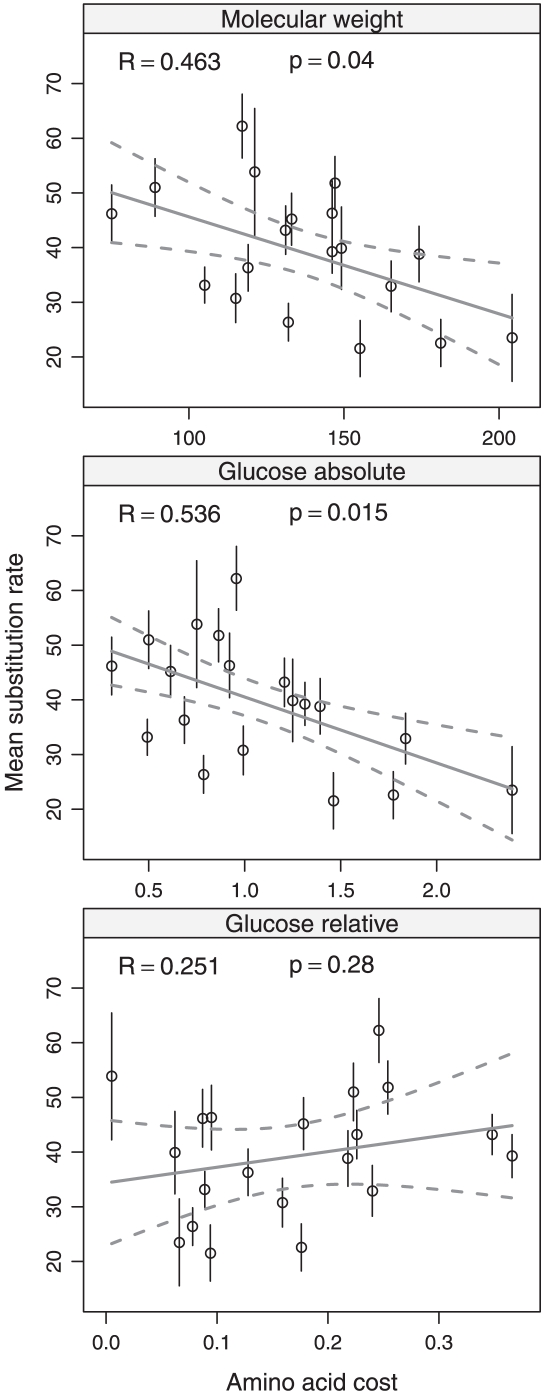
Comparison of amino acid substitution rate with biosynthetic cost. Each point is the mean substitution rate for one of the twenty standard amino acids. Substitution rates were estimated from alignments of *Saccharomyces* genes by Wall *et al.* [36]. Each amino acid substitution rate was normalised by tree length and then averaged across all alignment columns corresponding to the amino acid at that site in the ancestral protein sequence. Alignment columns containing gaps were excluded. The standard error of the mean for each amino acid substitution rate is shown as a bar in each point. Robust linear regression and 95% confidence intervals are used to indicated trend. Each plot indicates the Spearman's rank correlation between amino acid substitution rate and biosynthetic cost.

Table 3 shows the correlation of mean substitution rate with biosynthetic cost for all amino acid cost types. Figure 3 illustrates the relationship between mean substitution rate and cost for each amino acid for three different measures of biosynthetic cost that had a low pairwise Spearman rank correlation (see Table 2): molecular weight, 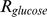, and 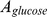. As predicted under the cost selection hypothesis, mean substitution rate is negatively correlated with some amino acid cost measures, including molecular weight, Akashi and Gojobori's energetic cost [2] and 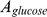 biosynthetic cost. However, the 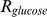 cost shows no correlation with mean substitution rate. Overall, 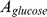 provides the best cost estimate to explain variation in substitution rates. These results suggest that cost minimisation may exert a selective pressure on rates of protein sequence evolution in *Saccharomyces* species and that amino acid cost derived using our systems biology approach is one of the best variables for explaining this trend. We hypothesise that this trend is due to expensive amino acids being conserved at certain sites due to selective constraints on structure of function, while the choice of amino acids in divergent sites is more affected by cost selection (see Discussion).

**Table 3:** Correlation between amino acid substitution rate and biosynthetic cost.

Cost type	*R*	*p*
*S. cerevisiae* A 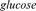	−0.54	0.015
*S. cerevisiae* R 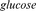	−0.25	0.28
Akashi & Gojobori (2002)	−0.47	0.034
Craig & Weber (1998) Energy	−0.28	0.23
Craig & Weber (1998) Steps	−0.19	0.43
Wagner (2005) Fermentative	−0.32	0.17
Wagner (2005) Respiratory	−0.4	0.077
Molecular Weight	−0.46	0.04

Each row represents the Spearman's Rank correlation between cost type and the mean substitution rate normalised by the estimated tree length of the encoding gene alignment for each of the twenty amino acids.

## Discussion

The principal outcomes of this work are twofold. First, we developed a novel method to estimate the cost of amino acid synthesis using a systems biology approach. Using FBA in the *S. cerevisiae* genome scale model we examined the sensitivity of different nutrient uptakes to changes in amino acid requirement. We compared our novel estimates of amino acid cost to six previously reported measures and showed that the absolute cost of amino acid biosynthesis under glucose limiting conditions (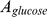) is highly correlated with previous cost measures, while relative cost under the same simulated environmental conditions (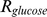) is not. We further showed that our systems biology approach can be applied to calculate environment-specific biosynthetic costs, which highlighted the effects of limiting factors in amino acid biosynthesis.

Secondly we investigated the predictive power of our biosynthetic cost measures in explaining variation in *S. cerevisiae* transcript and proteins levels, and explaining rates of amino acid substitution across four *Saccharomyces* species. Our analysis shows that biosynthetic cost does show an association with transcript and protein levels, but explains only a minor component compared with factors like codon usage bias that are related to translational optimisation. In contrast, we find that some amino acid cost measures are correlated with substitution rates in protein coding sequences.

### No single currency for amino acid biosynthetic cost

Our systematic comparison of biosynthetic costs described previously in the literature (Tables 1 and 2) shows that most cost measures are highly correlated with one another. Among previously reported measures, molecular weight is the least related (Figure 1), which is expected since the other cost estimates are based on manual curation of metabolic networks. This finding supports the view of Seligmann [7] that the molecular weight of an amino acid may account for biosynthetic investments not easily estimated from the metabolic network alone. Of the two costs estimated using FBA in glucose limiting conditions, our 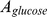 cost measure correlates with those previously described in the literature, confirming previous cost measures and validating our systems approach to estimating biosynthetic cost. One interesting point to note is that our 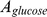 cost measure, like all previously reported cost measures (with the exception of Wagner's fermentative measure [5]), predicts tryptophan as being the most expensive amino acid to produce (Table 1). Tryptophan may be expensive because of its complex double ring structure and the number of high energy molecules required for its synthesis.

The 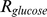 cost measure shows little relationship with any previously described cost metric under the same condition, and may provide a novel perspective on how to measure the cost of amino acid biosynthesis. The 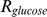 cost shows leucine and lysine to be the most expensive amino acids, whereas tryptophan is estimated as one of the cheapest in contrast to other previously reported cost measures (see above). Because our 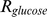 cost measure incorporates the amino acid requirement in the biomass reaction, this cost measure reflects the effect on nutrient uptake of a small relative increase or decrease as well as the usage of this amino acid in the cell. Therefore although a tryptophan molecule may be expensive to produce individually, its low relative usage makes it cheaper to maintain overall at the cellular level.

While it is clear that no single measure may fully capture all aspects of amino acid biosynthesis cost, we believe our systems biology approach has a number of advantages over previous methods. Given a genome-scale model, our computationally generated cost measures require no manual curation and allow cost calculations that are more explicitly replicable than other methods. Moreover, use of a computational model allows costs to be calculated under a different combinations of nutrient availability, permitting a more flexible approach to exploring costs under different cellular and environmental conditions. We also believe our approach takes into account a broader representation of cellular state, including all simulated reactions and metabolites, not just those in amino acid metabolism. Furthermore, as more information is included in genome scale models, the *in silico* predictions of amino acid cost may come to more closely represent their true costs *in vivo*. In particular the inclusion of thermodynamic constraints in the *S. cerevisiae* model, as has been done in *E. coli* [22], would be of particular relevance to explore in future work.

One drawback to our approach is that a species-specific stoichiometric model must be available to perform the analysis. This limitation may be important for relative costs, since they are more variable across species (Supplementary File S3), presumably because of their dependency on species specific amino acid usage in the biomass reaction, but less so for absolute cost estimates since they are highly correlated between divergent species such as *S. cerevisiae* and *E. coli*. A second point to consider is that the FBA estimated cost of an amino acid may be dependent on the objective function used in the model, For example, we assume that the *S. cerevisiae* growth strategy is to maximise biomass, or consider other strategies such as maximising ATP yield. Work by Schuetz *et al.* [23] suggests that the biological relevance of the FBA objective function is dependent on the environment considered, and research on this topic may present another avenue for further study.

A final consideration when using genome scale models is the method used to determine a flux solution. In this analysis we used FBA to find the flux distribution that minimised nutrient uptake. Other approaches however identify the solution that minimises the amount of metabolic adjustment from another flux solution [24] or minimising the number of on/off reactions between two solutions [25]. An exhaustive analysis of amino acid cost estimation using different optimisation methods may find differences in the costs estimated using a systems biology approach. Our analysis makes very small perturbations to biomass requirements and we anticipate that different model optimisation methods will have a much smaller effect on the model solutions compared with other applications such as complete *in silico* gene knockouts [26], [27].

### Translational optimisation over biosynthetic cost minimisation

In our analysis of the impact of cost on gene expression, multivariate regression models explained approximately 40% of variation in transcript and protein levels (Table 2). Of this variance in protein and transcript levels, the majority is explained by optimisation of the coding sequence for translation through codon usage bias rather than cost minimisation. This conclusion that biosynthetic cost may only be a weak selective force on *S. cerevisiae* gene expression is similar to recent results by Raiford *et al.* [6]. However, by examining cost minimisation in combination with features of translational optimisation such as codon bias, our analysis extends the work of Raiford *et al.* [6] to demonstrate that amino acid cost does appear to contribute a small effect on gene expression, independent of codon usage bias (Table 2, Figure 2). Our work also shows that the choice of cost type had only a small effect on the variation explained by the regression models, which may be expected given that the majority of the cost estimates are correlated.

### Costly amino acids evolve more slowly

Although biosynthetic cost may play only a minor role in terms of gene expression, we found a negative correlation between certain cost measures and rates of protein sequences evolution in yeast, in particular for molecular weight, Akashi and Gojobori's energetic [2] or 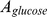 costs (Table 3, Figure 3). Our results suggest that expensive amino acids have a lower substitution rate and are more likely to be conserved while cheaper ancestral amino acids are more likely to be substituted even when controlling for the substitution rate of the encoding gene. These results support previous work in bacteria by Rocha and Danchin [28], who found that biosynthetic cost plays a small role in predicting substitution rates in *E. coli* and *B. subtilis* proteins, and by Hurst *et al.* [29], who found a negative relationship between the average cost per replacement and amino acid divergence. A possible hypothesis based on these trends is that expensive amino acids are only used for specific structural or functional roles and are therefore conserved, while cheaper amino acids may be under weaker structural or functional constraints and more likely to be substituted. In contrast to analysis of gene expression in the previous section, the trends observed between cost and substitution rate are more dependent on which cost type is used. Our results suggest that 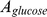 cost was one of the better measures for explaining variation in substitution rates (Table 3). Thus, a more detailed and wide ranging systems biology investigation of different environments may further help understand which type of amino acid cost and nutrient availability explain patterns of protein sequence evolution.

We are aware that the work presented here does not confirm cost selection as a causal factor in explaining the correlation between cost and rate of amino acid evolution, nor does it discount the possibility that biosynthetic cost may be correlated with other biochemical properties of amino acids that are thought to influence the pattern of protein sequence evolution [30]. A further point to consider is the method used to estimate the amino acid substitution rates. We used the codeml software [31] to provide a maximum likelihood estimate of amino acid substitution rate for each site. This method requires an *a priori* expectation of the relative rates of amino acid substitution in the form of a substitution matrix. Here we used the Whelan and Goldman (WAG) matrix [32], which was empirically estimated from observed amino acid substitution events in a curated set of homologs. Since differences in biochemical properties among amino acids are reflected in the relative rate of substitution among amino acids, it is possible that the correlations between cost and substitution rate we observe may be an artifact of the selective pressures that influence the substitution rates described in the WAG matrix as opposed to the direct effect of cost minimisation itself. We therefore attempted to control for this possibility by re-estimating amino acid substitution rates using a null rate matrix where rates of change between all amino acids were identical. Using substitution rates estimated from this null rate matrix, we observed the same trends between cost and substitution rate for molecular weight (Spearman 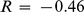, 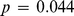), 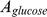 (Spearman 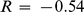, 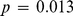) and 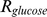 (Spearman 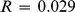, 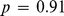). These results indicate that the correlation for either molecular weight or 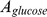 with amino acid substitution rates is not likely to be a spurious effect of other factors encoded by mutation bias or selection in the substitution matrix.

Thus we conclude that biosynthetic cost may play a role in yeast protein sequence evolution that should be considered alongside other factors that have been demonstrated to correlate with rates of amino acid substitution [33]. If cost minimisation does however prove to be a selective force on protein sequence evolution and amino acid biosynthetic costs vary considerably among groups or organisms we hypothesise that rates of protein sequence evolution may not follow universal trends across taxa. Moreover, it is clear that rates of amino acid substitution can provide a powerful filter for determining which measures of amino acid biosynthetic cost might be most biologically relevant for other analyses, such as effects on gene expression (see above). On these grounds, we may tentatively conclude that the 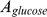 cost captures the *in vivo* cost of amino acid biosynthesis more accurately than other measures, and that our 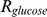 cost measure may provide the least biologically relevant representation of amino acid cost.

### Conclusions

We have developed a novel systems-biology approach to estimating the cost of amino acid biosynthesis and conducted a systematic investigation of how amino acid biosynthetic cost has shaped gene expression and protein evolution in yeast. Our analysis indicates that amino acid biosynthetic cost plays a limited role in transcript and protein production relative to what might be expected, given that a predicted 80% [5] of the cellular ATP budget is devoted to protein synthesis. Our results do reveal a negative correlation with amino acid biosynthetic cost and rates of amino acid substitution in *Saccharomyces* species, which may highlight an important selective force governing molecular evolution in yeast.

## Materials and Methods

### Simulating a genome scale model of metabolism

Each *S. cerevisiae* and *E. coli* genome scale model is a matrix detailing the stoichiometry of a set of metabolic reactions representing the ratios of metabolites consumed and produced by each reaction. A species genome scale model matrix *S* is size 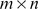 where *m* is the total number of metabolites and *n* is the total number of reactions. The position 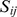 in the matrix represents the coefficient of metabolite *i* in reaction *j*. A positive coefficient indicates the metabolite is produced, while a negative value indicates the metabolite is consumed. A value of 0 means the metabolite does not participate in the reaction.

Flux balance analysis of a genome scale model aims to solve the equation 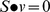 using linear programming, where *v* is a vector of predicted flux distributions (i.e. reaction rates) and is equal in length to the number of reactions in the model. Multiple solutions may exist for *v* and a biologically meaningful reaction is usually optimised such as production of biomass or ATP. Additional constraints may be placed on the model solution, for example that a certain reaction flux may not be negative which then forces the reaction to only proceed in the forward direction. Constraints on the reactions that transport nutrients in and out of the model can be used the simulate different combinations of nutrient availability.

### A systems biology approach to estimating the cost of amino acid synthesis

Flux balance analysis was performed using the COBRA toolbox [34] and the lpsolve library [35], running in the MATLAB environment. The genome scale models used were iND750 for *S. cerevisiae* [26] and iJR904 for *E. coli* [12]. Reaction fluxes are measured in mmol of metabolite, per gram dry weight of biomass, per hour (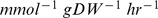). Metabolite concentrations are measured in mmol of nutrient per gram of dry weight biomass (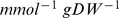). The rate of the biomass production reaction, synonymous with cellular growth, is measured per hour (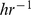).

For each of the twenty amino acids in the model biomass reaction we increased or decreased the stoichiometric requirement of the amino acid at position 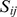 where *i* is the amino acid and *j* is the biomass reaction. The change in amino acid requirement ranged from 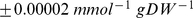 for absolute estimations of amino acid cost and 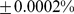 for the relative estimates of cost.

For each change in amino acid requirement, biomass production flux was fixed and the model solved to minimise the flux entering the cell for one of three nutrients: glucose, ammonia, sulphate. The other nutrient entry reactions in the model (water and oxygen) were not considered as objective functions. Each nutrient transport reaction was set to have a lower boundary of 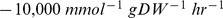 to effectively make the allowable entry of the nutrient into the cell limitless. When estimating a cost for a given nutrient it is important to note there were no other equivalent nutrient sources entering the cell. For example, glucose transport was the only source of high energy sugar and ammonia was the only source of nitrogen.

Using data points produced from the above series of FBA simulations, the absolute cost for each amino acid was derived as the slope between the absolute change in amino acid requirement and the corresponding effect on nutrient uptake flux. Likewise, the relative cost was estimated from the percentage change in amino acid requirement and the effect on nutrient uptake flux. As noted above, the amino acid requirement for growth is defined as millimoles per gram of dry weight biomass (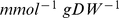) and the supply of nutrients into the cell is defined as the per hour entry of each molecule (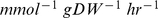). Thus, the units of absolute cost are per hour change (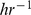) and the units of relative cost are 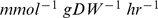.

All costs were estimated at a range of growth rate fluxes: 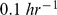, 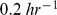 and 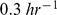. After estimation each cost was rescaled by dividing by the growth rate at which it was estimated. Dividing each cost by the growth rate changed the units of our cost measures, resulting in the relative costs becoming 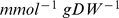 and the absolute costs becoming unitless. The MATLAB code used for the estimation of amino acid cost is available in Supplementary File S7. The R code used to compare the different amino acid cost types in Figure 1 is available in Supplementary File S8.

### Relationship between absolute and relative cost estimates

The absolute cost estimates can be defined mathematically as the differential between changes in amino acid requirement (*x*) and the corresponding effect on nutrient uptake (*U*). Shown in the following equation:
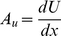


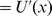



The relative estimate of amino acid cost can be defined as the differential of a percentage change in amino acid requirement (*r*) and the corresponding effect on nutrient uptake (*U*). Shown in the following equation:
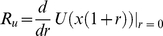


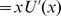



These two equations illustrate that an absolute estimate of amino acid cost can be scaled to a relative cost through multiplication by *x* representing the stoichiometry of the given amino acid in the biomass reaction. This reverse is true where a relative cost can be scaled to an absolute cost by division. In addition to showing this theoretically we also demonstrated these results empirically (results not shown).

### Determination of transcript and protein characteristics

The Codon Adaptation Index (CAI) for each *S. cerevisiae* gene was taken from Wall *et al.* 2005 [36], and the number of genomic tRNA copies for each amino acid was taken from Akashi [15]. The total number of tRNAs were summed over the length of the protein, and then divided by the length of the protein to give a mean number of tRNAs per residue for each gene. Previously reported amino acid biosynthetic costs were obtained from Craig and Weber [1], Akashi and Gojobori [2], Wagner [5] and Seligmann [7]. For each gene, the average tRNA gene number, biosynthetic cost, or atomic content was computed as the sum of the count or cost over the encoded protein divided by the length excluding stop codons. Prior to regression, each of these variables was transformed by the natural logarithm then scaled to have the same mean and variance. Scaling was performed by subtracting the mean then dividing by the root mean square for each variable. The aim of this was to reduce any over-variation and heteroscedasticity biasing fit estimation.

### Modelling the effects of biosynthetic cost on gene expression

Multiple regression was used to measure the importance of biosynthetic cost on transcript and protein expression using the R statistical computing language [37]. The measured quantities of either transcript or protein levels were treated as the response variable and biosynthetic cost, CAI and atomic content were used as explanatory variables. Atomic content consisted of three independent variables: carbon, nitrogen and sulphur content. Experimental conditions that differed among replicates were treated as fixed effects in the regression, and included as interaction terms. Initially, all possible interaction terms were considered and automated step-wise regression used to remove superfluous interaction terms based on a penalised log-likelihood score – Akaike's Information Criterion (AIC) [18].

To estimate the importance of each of the equation parameters, the data was regressed without the variable in question, and then compared to the regression containing all terms, again using AIC. For example, to estimate the importance of nitrogen in the Castrillo *et al.* 2007 [11] transcript data, the regression was first fitted using all factors - environment, dilution rate, CAI, tRNA gene number, biosynthetic cost, nitrogen, carbon and sulphur content. The importance of nitrogen was then determined by repeating the regression fitting with the same variables except nitrogen content. The contribution of nitrogen content to explain the variation was then estimated from the difference in the regression without nitrogen compared with the regression containing all terms. This process was performed for all factors in the equation, and then repeated for all biosynthetic cost estimates as the cost variable in the equation. The R code used to plot the the regression results in Figure 2 is available in Supplementary File S8.

### Gene expression data

The experimental transcriptomic data used in this analysis are from Castrillo *et al.* 2007 [11] and the proteomic dataset was produced by Ghaemmaghami *et al.* 2003 [21]. Briefly, the Castrillo *et al.* 2007 [11] experiments continuously cultured *S. cerevisiae* using a chemostat under four nutrient limiting conditions and three dilution rates, for a total of twelve different experimental conditions. Transcript levels were estimated from replicate microarray analysis of total RNA which was then processed by robust multi-array (RMA) quantile normalisation [38]. The tabulated transcript data used in this analysis are available in Supplementary File S4.

Protein data produced by Castrillo *et al.* [11] measured up/down regulation of a protein against a background, which is not suitable as a measure of absolute protein expression levels. Therefore, we used data from Ghaemmaghami *et al.* 2003 [21] for our analyses of cost in protein production. The protein data was produced from tandem affinity purification (TAP) of TAP-tagged *S. cerevisiae* ORFs. Expression levels for each protein were determined using antibody-tag based quantification. These data were converted to absolute protein molecules per cell using a purified *E. coli* INFA-TAP construct standardised against the range of yeast TAP tag protein observations. The tabulated protein data used in this analysis are available in Supplementary File S5.

For the model analysis, protein levels were transformed by the natural logarithm then scaled. Transcript levels were scaled, but not log transformed as this was done in the original processing.

### Estimation of amino acid substitution rates

Codon-based nucleotide alignments of coding regions from orthologs of *S. cerevisiae*, *S. mikatae*, *S. bayanus*, and *S. paradoxus* genes were taken from Wall *et al.* [36]. Alignments containing less than four species or where the *S. cerevisiae* sequence did not match the SGD reference sequence [39] were ignored.

The relative substitution rate and ancestral state of each alignment column was estimated using codeml [31] with the Whelan and Goldman (WAG) amino acid substitution rate matrix [32]. The codeml parameters used were as follows: fix_kappa, 0; seqtype, 3; aaDist, 0; Malpha, 0; kappa, 2; cleandata, 0; ncatG, 8; model, 3; method, 0; fix_omega, 0; getSE, 0; RateAncestor, 1; omega, 0.4; NSsites, 0; verbose, 1; fix_blength, −1; icode, 0; fix_alpha, 0; CodonFreq, 2; alpha, 0.5; Mgene, 0; clock, 0. The Newick representation of species tree used was: ((*S. cerevisiae*, *S. paradoxus*) *S. mikatae*, *S. bayanus*).

The substitution rate for each alignment column was divided by the codeml estimated alignment tree length to control for the mutation rate of the encoding gene. The single gene alignment with an estimated tree length of 0 was removed from the data. The ancestral amino acid at each alignment column was inferred and the substitution rate of all alignment columns was averaged over all sites where the same ancestral amino acid was observed. Sites that contained a gap in the any of the descendant sequences were ignored. The tabulated substitution rate data used in this analysis are available as Supplementary File S6. The R code used to plot substitution rate versus biosynthetic cost in Figure 3 is available as Supplementary File S8.

## Supporting Information

File S1 Amino acid costs. Amino acid cost estimates used in the analysis.
(0.00 MB ZIP)

File S2 Amino acid cost correlations. Spearman's rank correlations between cost estimates. The first 18 rows are R values, and the latter 18 rows are p values.
(0.00 MB ZIP)

File S3 Comparison of the genome scale model derived cost data sets. Comparison of FBA estimated amino acid cost with Akashi and Gojobori cost (left), and molecular weight (right). Both *S. cerevisiae* and *E. coli* measures are included to illustrate correlation of cost estimates between species. Estimated cost values have been rescaled around their mean value to allow comparisons across species. Trend lines are indicated using “loess” smoothing.
(0.02 MB EPS)

File S4 Tabulated transcript data set. The transcript data from Castrillo *et al.* 2007 tabulated with cost, atomic composition, tRNA gene number and CAI.
(5.68 MB ZIP)

File S5 Tabulated protein data set. The protein data from Ghaemmaghami *et al.* 2003 tabulated with cost, atomic composition, tRNA gene number and CAI.
(0.68 MB ZIP)

File S6 Tabulated amino acid substitution rate data. Substitution rate estimates at each position in 3334 of the Saccharomyces species alignments produced by Wall *et al.*
(4.20 MB ZIP)

File S7 MATLAB code to estimate amino acid cost. The MATLAB and COBRA code used to estimate amino acid cost.
(0.01 MB ZIP)

File S8 The R code used to generate the figures presented in this work.
(7.94 MB ZIP)
