# Correction: Evolutionary Systems Biology of Amino Acid Biosynthetic Cost in Yeast

**DOI:** 10.1371/annotation/b60feca4-9a4f-4311-8a07-4bf16a2f9316

**Published:** 2010-10-08

**Authors:** Michael D. Barton, Daniela Delneri, Stephen G. Oliver, Magnus Rattray, Casey M. Bergman

This article was republished on September 20, 2010 because an earlier version of the manuscript was published with incorrect data. In the correct version, amino acid substitution rates were normalised by the encoding gene tree length. The subsequent correlations between amino acid costs and substitution rate were smaller, but still significant and do not change the main conclusion of the manuscript, that amino acid substitution rates show a trend with biosynthetic cost. 

